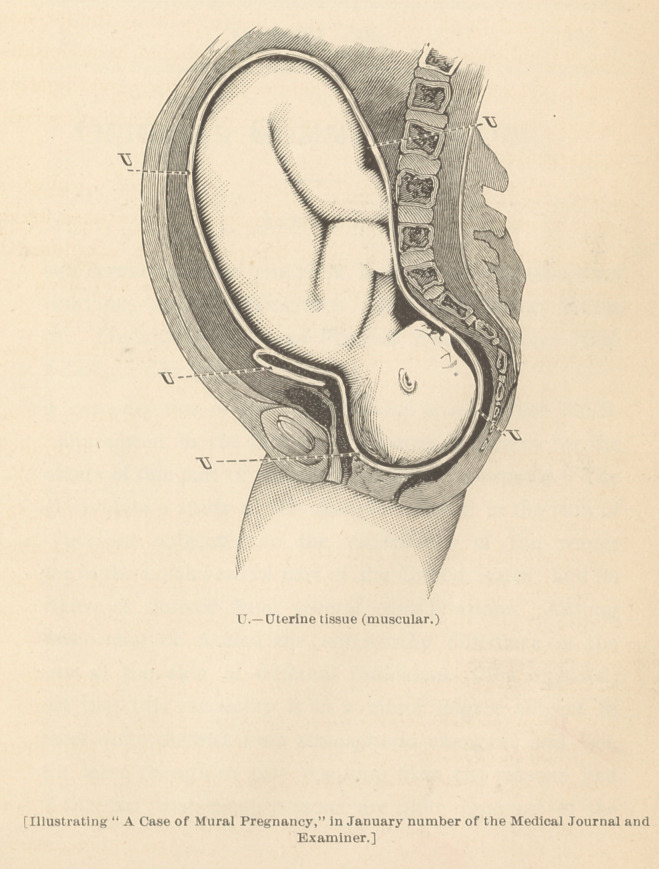# Chicago Gynæcological Society

**Published:** 1885-02

**Authors:** 

**Affiliations:** 2330 Indiana ave.


### SOCIETY PROCEEDINGS.

---

CHICAGO GYNÆCOLOGICAL SOCIETY.—*Interesting Case of Double Ovariotomy.* DR. E. C. DUDLEY.

*Regular Meeting, Friday, Jan. 16th, 1885.*—The President, Dr. H. P. Merriman, in the chair.

Dr. E. C. Dudley read a paper on an interesting and unusual case of double ovariotomy. The specimens were subsequently exhibited.

The patient, eighteen years old, unmarried, came to Mercy Hospital about eighteen months ago, to consult Dr. Dudley about marked abdominal enlargement. The diagnosis of monocystic tumor of the parovarium, or broad ligament, was made. The cyst was aspirated, and two gallons of fluid were removed. This fluid possessed the following characters: sp. gr., 1000.5; neutral reaction; limpid as water; odorless; colorless; on microscopical examination, no morphological constituents were detected; on chemical examination, certain mineral salts were found, but no albumen.

The patient experienced so much relief, after aspiration, that she left the hospital with the impression that she was permanently restored to health.

About three months ago she visited Dr. Dudley at his office. It was found that the cyst was partially refilled. An operation was determined upon.

The preparatory treatment of the patient,—apart from tonics, the most nutritious of foods, frequent baths, and finally a Turkish bath, immediately preceding the day of operation,—consisted in the exhibition of remedies designed to increase the tonicity of the muscular coats of the intestines, and to expel all gases. For this purpose, the patient was given, two or three times daily, a mixture of columba, rhubarb and compound tincture of cardamom. In a case operated upon early in the autumn, Dr. Dudley had experienced much difficulty in the management of the intestines, which were distended with gas. In this case, subjected to the preparatory treatment just detailed, absolutely no difficulty in that direction was encountered.

The *mons veneris* was shaved, the vagina irrigated immediately before operation. The details of rigidly antiseptic surgery were observed with reference to the operating room, instruments, operator and assistants.

On Oct. 29th, Dr. Dudley, assisted by Dr. W. W. Jaggard, Dr. R. W. Bishop and Dr. W. E. Casselberry, performed ovariotomy, and removed both ovaries and tubes, together with a large monocyst of the left broad ligament. The pedicle of the large cyst was very vascular, and after transfixion with the passage of the ligature around each half, the operator was compelled to ligature *en masse* below the point of transfixion. The pedicle was afterwards seared above the Baker-Brown clamp with Paquelin's thermo-cautery.

Ether was employed as the anæsthetic.

The patient reacted well. At noon, two hours after operation, temperature 100.7°; pulse, 150; respiration, 20. She complained of nausea. At night, temperature, 100.8°; pulse, 115; respiration, 20; nausea continues; no tympanites.

Oct. 30th. Morning; temperature, 100.4°; pulse, 112; respiration, 20. Evening, temperature, 101°; pulse, 124; respiration, 20.

Throughout the day the patient moaned and tossed, complained of nausea, vomited incessantly. A peculiar talkative delirium ensued. Morphine and atropine were given to suppress vomiting; discontinued the atropine, fearing its cerebral action. Hot water, as suggested by Mr. Keith, was tried, with hope of checking nausea and vomiting, without success. The deodorized tincture of opium was substituted for morphia. The talkative delirium, nausea and vomiting continued unabated. The patient retained only a little ice water at long intervals.

Oct. 31st. Morning; temperature, 99.8°; pulse, 110; respiration, 20. Evening; temperature, 99.8°; pulse, 110; respiration, 20.

Nervous symptoms, greatly exaggerated; nausea and vomiting unabated; pain in the abdomen complained of at intervals. Codeia was substituted for the other opiates. Small quantities of milk and lime water, at intervals, were exhibited. The patient remained in the same condition. Talkative delirium, no sleep, nausea and vomiting, pain in the abdomen at intervals; no tympanites.

Nov. 1st. Morning and evening temperature, 99.7°; pulse, 106; respiration, 20. Talkative delirium, nausea, vomiting of bile,—the "mouth-filling" of Mr. Keith,—abdominal pain, no tympanites. She commenced to menstruate, or at least blood began to escape from the vagina.

Nov. 2d. Morning; temperature, 98.8°; pulse, 114; respiration, 18. Evening; temperature, 98.6°; pulse, 135; respiration, 21.

Nervous symptoms more distressing; nausea and vomiting continuing; patient becoming emaciated; face taking on a pinched, anxious expression. Valentine's beef juice exhibited; mustard to epigastrium; strychnia, in small doses; champagne; bisulphate of quinine, *per rectum.*

Nov. 3d. Morning; temp., 101°; pulse, 130. 10 A. M., temp., 101.2°; pulse 160. 10:30 A. M., temp., 101.6°; pulse, 178.

Persistence of wild, talkative delirium, nausea, vomiting, abdominal pain. The pulse, while very rapid, was not the feeble pulse of collapse. Dr. Dudley had one case of peritonitis following perforation of the posterior uterine wall, in Mercy Hospital, that died of septic poisoning, although the temperature did not rise above 100°.

With the aid of Dr. R. W. Bishop, Dr. Dudley etherized the patient, removed four or five stitches from the lower end of the abdominal incision, and inspected the cavity of the abdomen. The peritoneum was examined; no lymph was noticed; no gas in the intestines. The pedicles were not covered with lymph. A disinfected sponge, on a holder, was passed into the *cul-de-sac* of Douglas; about one-half of a fluid ounce of bloody serum was removed. This red stained fluid was odorless; it was not further examined. Feeling no good had been accomplished, the incision was reunited. This operation was performed at 10:30 A. M.

Temperature at 12 M., 101°; pulse, 150.

" at 2 P. M., 100°; pulse, 144.

" at 3 P. M., 100°; pulse, 135.

" at 7 P. M., 100°; pulse, 140.

Jactitation, wild delirium; nausea and vomiting; pain in the region of the coeliac axis; no tympanites. Rectal alimentation.

Nov. 4th. Morning; temperature, 100°; pulse, 135-150. Whisky, beef tea and milk at regular intervals; bisulphate of quinine *per rectum*. Persistence of the symptoms before detailed.

In the afternoon, the tongue was dry, cracked, ready to bleed; decidedly less nausea and vomiting. The nurse was directed to give the patient an enema of soap and water, with extract of ox-gall. One quart of this mixture was injected into the bowel. No evacuation of the contents of the rectum following, a tube was introduced, which was followed by that classical sign, the audible escape of flatus. Mr. Keith says that if the intestines have sufficient muscular energy to expel flatus, the prognosis becomes more favorable. It is probable a reversed peristalsis occurred, and the injection was entirely absorbed. One hour afterwards the tongue became moist. Evening temperature, 101° ; pulse, 135.

Acting on the suggestion from the enema, one quart of strong beef tea was introduced into the bowel.

Nov. 5th. Patient evidently in better condition. Temperature, morning and evening, 98-99°; pulse, 120-130. One quart of strong beef juice was exhibited *per rectum*, and carried well up into the bowel. About three-fourths of this quantity was retained. Slight nausea and vomiting. Bowels were moved spontaneously, with evacuation of a large quantity of dark, tarry, fœtid fæces. The sutures, originally inserted, were removed.

Nov. 6. Morning; temp., 98.5°; pulse, 90.
Evening; " 98.1°; pulse, 115.

Bowels were again moved spontaneously, with evacuation of dark, tarry, fœtid fæces; all the symptoms of the patient assuming a favorable character. Beef-tea, whisky, quinine, and nutrient enemata continued. From this time on, the patient made an uninterrupted recovery. At the end of two weeks she was removed to St. Luke's Hospital. She is now in good health and pursuing her usual avocation.

In conclusion, Dr. Dudley called attention to the three following subjects, and requested that the discussion should be more particularly limited to their consideration..

I. *Preparatory Treatment of the Intestinal Tract.*—Is it possible to render the intestines manageable during an operation in the abdominal cavity by any dietetic or medical agencies? The escape of the intestines without the abdominal parietes was a very distressing complication. The shock of the operation was increased, large vascular areas were rapidly cooled, notwithstanding the fact that they might be enveloped in warm, disinfected fabrics. It was not always easy to return the intestines to the cavity of the abdomen, and even then there was danger of strangulation. He was of the opinion that, in the case reported, the rhubarb, columbo and cardamom were active in restoring tonicity to the muscular coats, and in the expulsion of flatus.

II. *The Retention of Enemata.*—It was a matter of surprise to him that one quart of fluid exhibited *per rectum*, on three successive days, should be retained. He supposed that the liquid portions of the injections were absorbed at once, in consequence of the state of the tissues, resulting from exhaustion. The return of the tongue to a moist condition, within a very short space of time, immediately following the injection, was evidence in favor of this explanation.

III. *The Re-opening of the Abdominal Incision.*—Had it done any good? What produced the excessive nervousness? What was the cause of the nausea and vomiting?

The problem was an intricate one. It has been asserted that a very tight ligature around the pedicle can act as a reflex irritant. The sutures in the abdominal incision are sufficient, at times, to produce various obscure reflex symptoms. He had one case—his first case—in which uncontrollable vomiting yielded immediately upon the withdrawal of a rubber drainage tube.

It may have been that the case reported was just on the eve of recovery, when the abdomen was reopened.

The fluid removed was small in quantity, and without odor. The peritoneum, however, can secrete with wonderful rapidity, and then absorb the secretion. He had not examined the fluid microscopically or chemically. He had dusted into the *cul-de-*

*sac* a small quantity of iodoform. Whether *post hoc* or *propter hoc*, the patient recovered.

If it was a case of exhaustion, the beef, milk, and whisky deserved the credit. If a case of sepsis, the reopening of the incision was the potent factor. In answer to a question by Dr. Sawyer, Dr. Dudley said, that although the delirium of the patient was attended with visions of snakes, alcohol was out of the question, from his own knowledge of the habits of the patient. In answer to Dr. Merriman's question as to the preparation of the ligatures, Dr. Dudley said that silk thread was used exclusively for sutures and ligatures. This silk thread was boiled for ten minutes in 95 per cent. sol. carbolic acid; then for thirty minutes in 5 per cent. sol carbolic acid; finally it was deposited in a solution of the bichloride of mercury, one to four thousand.

Dr. Dudley then exhibited the specimens. The right ovary was slightly enlarged, and in the commencing stage of cystic degeneration. A cyst, about the size of a hickory nut was found in the right broad ligament. The left ovary was converted into a mass of fibrous tissue, about the size and shape of a kidney. Springing from the left parovarium or from the left broad ligament was the large monocyst, to which allusion has been made. This cyst, at the time of operation, contained about forty pounds of fluid.

*Discussion.*—Dr. Henry T. Byford thought that the reopening of the abdominal incision was unnecessary. Relief could not have come from the removal of such a small quantity of fluid as one-half fluid ounce. The improvement, immediately following the operation, was probably due to the stimulant effect of the ether. The delirium seemed to him to be that of alcohol or cerebral anæmia. He did not think that the symptoms of nausea and vomiting could be explained by any local irritant such as ligatures or sutures. It was a case of exhaustion, cured by the judicious exhibition of beef, whisky and milk.

Dr. Edward Warren Sawyer said the patient owed her life to the persistent bravery of the operator, and congratulated him upon his success. He was surprised to hear that no albumen was detected in the aspirated fluid. Friedrichs says albumen is always present in such cases, but absent in echinococcus cysts.

Dr. Flavius M. Wilder said that egg albumen in water was frequently tolerated by the stomach when other matters were rejected.

Dr. W. W. Jaggard thought the secretory and resorptive functions of the peritoneum were matters of positive knowledge. Dr. Anton Wölfler, in a recent paper, has taken, substantially, Dr. Dudley's position. It is quite possible that the one-half fluid ounce of bloody serum, found in the *cul-de-sac*, represented the ultimate stage of resorption of a much larger quantity of fluid.

One-half fluid ounce of fluid, however, may contain enough sepsin or sufficient bacteria to produce the most fatal pyæmia. There is no quantitative relationship in regard to the virulency of certain poisons.

The mere reopening of the abdominal incision seems to act, under certain conditions, in a favorable manner. The reason is unknown. It is an empirical fact, acknowledged by a number of leading surgeons.

He thought Dr. Dudley deserved great credit for his action, in taking up the suggestion of numerous operators, when he knew positively no foreign body was contained within the abdominal cavity.

Dr. W. E. Casselberry wished to emphasize the importance of the preparatory treatment of the intestinal tract. He had been present at both of the operations, to which Dr. Dudley had referred, and was struck by the difference in the behavior of the intestines. The mixture employed by Dr. Dudley resembled a favorite formula of the late Dr. Geo. B. Wood. This formula was advised for the expulsion of flatus and restoration of tone to the intestinal muscular walls.

Dr. D. T. Nelson said it was highly important to avoid the use of opium, when it was possible. The less opium, in general terms, the less the tendency to vomiting. As to the pedicle, he entertained grave doubts as to the propriety of passing the ligature *en masse.* In strangulated hernia, uncontrollable vomiting frequently resulted from the inclusion of omentum in the ligature. Would it not be better to open the pedicle, and pass a ligature around each vessel separately? He thought Bantock, Thornton and Spencer Wells advised the same treatment as in the securing of the vessels in an amputated leg.

Dr. Sawyer said he had seen Dr. John E. Owens remove a testicle; following the passage of the ligature around the cord, uncontrollable vomiting occurred.

The reopening of the abdominal incision was looked upon with too much fear. He had been present at an operation in which all the blood had not been removed from the *cul-de-sac;* the patient died of septicæmia. At the autopsy, Douglas's pouch was filled with blood. An operation might have saved life. Another case in point, was one of normal, double ovariotomy. After the operation, the patient sank rapidly. The operator concluded the patient was suffering from internal hæmorrhage. Twelve hours later, the abdominal incision was reopened, and after two hours search the bleeding vessels were secured. The patient recovered.

Dr. H. P. Merriman endorsed Dr. Dudley's reopening of the abdominal incision in the case presented.

In regard to the preparatory treatment of the intestinal tract, he was not certain that the exhibition of columbo, rhubarb, and cardamom had produced the favorable result in the one case, nor that the neglect of preparatory treatment was operative in the troublesome condition in the other. He did not think the opium was the cause of the vomiting, since the nausea disappeared while the patient was still under the influence of opiates.

He did not think the ligature *en masse* or the clamp could be replaced by the method suggested by Dr. Nelson. The danger from hæmorrhage was too great. It was an admirable subject for study. If the pedicle was complely destroyed the danger of pinching nerve filaments would be less.

The quantity of fluid removed after reopening the incision was not sufficient to account for the improvement in symptoms.

Dr. Nelson referred to the compression forceps, and ligature placed above, as designed to obviate the inclusion of nerves, still sensitive.

Dr. Dudley closed the discussion. The mere opening of the abdomen seemed to act in a remarkable manner under certain conditions. Tubercle of the peritoneum, papilloma of the omentum, were pathological states which had been influenced favorably by the procedure.

*Cocculus indicus* is supposed to have the same effect to prepare the intestinal tract as the mixture exhibited.

It was not necessary that a discharge should have a foul odor or be large in quantity, in order to be capable of producing pyæmia.

The Society adjourned to meet at the Palmer House, as the guests of Dr. Sawyer and Dr. Jaggard, on Friday evening, 20th Feb., 1885. Dr. Christian Fenger will present a report of the result of his anatomical investigations of Professor Byford's "Two cases of mural pregnancy."

Dr. H. T. Byford will read a paper on "The Functions of the Membranes in Labor."

The inaugural thesis of Dr. Chas. Caldwell on "Two Interesting Cases in Obstetrics" will be discussed.

W. W. JAGGARD, *Editor*,

Jan. 19th, 1885. 2330 Indiana ave.

## Figures and Tables

**Figure f1:**